# Functional lncRNA-miRNA-mRNA networks in rabbit carotid atherosclerosis

**DOI:** 10.18632/aging.102778

**Published:** 2020-02-11

**Authors:** Yingnan Wu, Feng Zhang, Rui Lu, Yanan Feng, Xiaoying Li, Shuang Zhang, Wenying Hou, Jiawei Tian, Xianchao Kong, Litao Sun

**Affiliations:** 1 Department of Ultrasound, The 2nd Affiliated Hospital of Harbin Medical University, Harbin 150081, Heilongjiang, China; 2 Department of Ultrasound, The First Affiliated Hospital of Xiamen University, Xiamen 361003, Fujian, China; 3 Department of Ultrasound, Xuanwu Hospital Capital University, Beijing 100053, China; 4 Department of Gynecology and Obstetrics, The 2nd Affiliated Hospital of Harbin Medical University, Harbin 150081, Heilongjiang, China; 5 Department of Ultrasound, Shenzhen University General Hospital, Shenzhen 518055, Guangdong, China

**Keywords:** atherosclerosis, lncRNA, ceRNA, network, rabbit carotid arteries

## Abstract

Atherosclerosis is one of the most common clinical cardiovascular disorders. Accumulating evidence indicates that lncRNAs exert critical functions in atherosclerosis; however, their functional roles and regulatory mechanisms remain unclear. In this study, we induced atherosclerotic plaques in three rabbit carotid arteries through an atherogenic diet and balloon injury; three age-matched rabbits were fed normal chow and served as controls. We thoroughly investigated the RNA (mRNA, lncRNA and miRNA) expression profiles in atherosclerotic rabbit carotid models with deep RNA sequencing. We identified several significantly differentially expressed RNAs. The corresponding lncRNA-miRNA-mRNA network was constructed, and the significantly dysregulated network was selected. Furthermore, Gene Ontology and  Kyoto Encyclopedia of Genes and Genomes analyses indicated that the mRNAs in the network were involved in leukocyte activation, cell proliferation, cell adhesion molecules and cytokine-cytokine receptor interaction. After rigorous screening, we obtained a differentially expressed lncRNA-miRNA-mRNA interaction network associated with atherosclerosis. In the network, *XLOC_054118* and *XLOC_030217* upregulate the *CHI3L1*, *SOAT*, *CTSB* and *CAPG* genes by competitively binding to the miRNA *ocu-miR-96-5p*. *XLOC_062719* and *XLOC_063297* upregulate *CTSS*, *CTSB* and *EDNRA* genes by competitively binding to the miRNA *ocu-miR-185-5p*.

## INTRODUCTION

Atherosclerosis (AS), a chronic disorder affecting the blood vessel walls, is characterized by an imbalance between the inflammatory response and lipid metabolism [1]. AS is the common pathological basis of multiple cardiovascular and cerebrovascular disorders and has become the main cause of mortality and long-term morbidity worldwide [2]. Currently, AS medications primarily reduce plasma cholesterol concentrations and blood pressure, effectively reducing tissue damage caused by the disease [3]. However, AS-related mortality and morbidity remain high. Genetic factors represent a major determinant of AS risk [4]. Thus, there is a need to identify the molecular pathways responsible for AS development and to develop valuable therapeutic medicines and prognostic biomarkers for AS.

Long noncoding RNAs (lncRNAs) were recently recognized to be broadly transcribed in various eukaryotic genomes ranging from those of nematodes to those of humans [5]. LncRNAs comprise transcripts > 200 nucleotides in length and regulate many biological mechanisms [6], such as genomic imprinting and chromatin modification [7]. Additionally, accumulating evidence has indicated that lncRNAs have extensive and complex impacts on the development and progression of AS. For example, the expression levels of the lncRNAs *Zfas1, SNHG6* and *GAS5* distinctly increased in individuals with atherosclerotic plaques compared with the expression levels in normal controls [8]. Li et al. [9] found that lncRNAs regulated AS-related processes in endothelial cells, macrophages, smooth muscle cells, and lipid metabolism. However, the roles of lncRNAs and their functions in AS have mostly been unreported. Salmena et al. [10] suggested a complicated posttranscriptional gene expression regulatory network termed competing endogenous RNAs (ceRNAs), wherein circular RNAs (circRNAs), lncRNAs, and other noncoding RNAs act as molecular sponges to inhibit mRNA function by sharing at least one microRNA (miRNA) recognition element (MRE). LncRNAs with similar sequences to targeted miRNAs serve as ceRNAs to modulate the level of protein-coding genes and to modulate cell biology by sponging miRNAs [11]. LncRNA-related ceRNAs serve as novel posttranscriptional regulators and have been reported to have crucial roles in various disorders. The lncRNA *XIST* was markedly overexpressed in gastric carcinoma and acted as a ceRNA to meditate *EZH2* expression by naturally sponging miRNA *101* [12]. The lncRNA *HOST2* functioned as a molecular sponge of the miRNA *let-7b* to downregulate *let-7b* expression, consequently affecting epithelial ovarian carcinoma [13]. However, the mechanisms of lncRNA-related ceRNA in AS remain largely unknown. Therefore, it is necessary to study lncRNA-miRNA-mRNA competitive regulatory networks to comprehensively understand the impact of ceRNA crosstalk on AS.

In this study, miRNA, mRNA and lncRNA expression profiles were constructed in carotid atherosclerotic rabbit models with deep RNA sequencing (RNA-seq). Furthermore, we investigated differentially expressed (DE) profiles of miRNA, mRNA and lncRNA in AS and detected lncRNA-miRNA-mRNA networks in carotid atherosclerotic rabbit models. Gene Ontology (GO) and Kyoto Encyclopedia of Genes and Genomes (KEGG) analyses were performed to explore the potential regulatory functions of lncRNA. Then, functional lncRNAs were detected in lncRNA-miRNA-mRNA networks with the highest function in AS. Our results provide valuable resources to develop therapeutic pharmaceutical targets and molecular diagnostic tools.

## RESULTS

### Establishment of atherosclerotic rabbit carotid artery models

**Figure 1:**
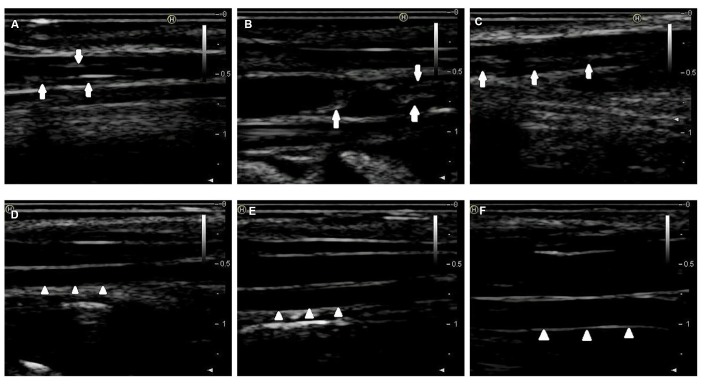
**2D-ultrasound images of rabbit carotid arteries at the 12^th^ week.** (**A**–**C**) 2D-ultrasound images reveal that obvious atherosclerotic plaques formed on rabbit carotid arterial intima as the arrows show. (**D**–**F**) 2D-ultrasound images demonstrate that carotid arteries intima of rabbits treated with normal chow still maintain smoothness as the arrows show.

**Figure 2:**
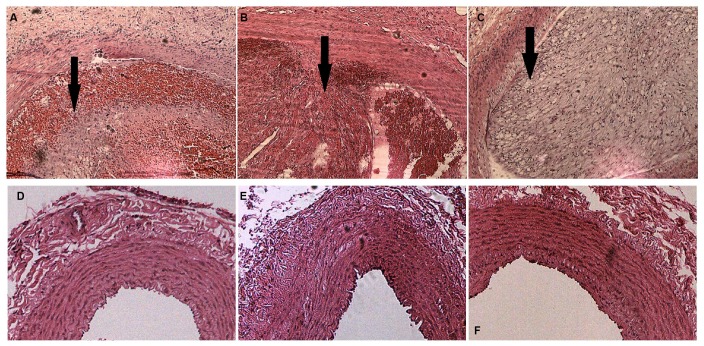
**H&E staining of rabbit carotid arteries.** (**A–C**) H&E-stained vasculature shows plaques of varying degrees in rabbit carotid arteries in the AS group as the arrows show, ×40. (**D**–**F**) H&E-stained arteries show that no evident abnormal changes are detected in rabbit carotid arteries of the case group, ×40.

By combining endothelial injury and an atherogenic diet, we thickened and roughened the intima and induced multiple irregular hypoechoic plaques in rabbit right common carotid arteries as indicated by two-dimensional (2D) ultrasound at the 12th week (Figure 1A–1C). In contrast, the carotid artery intima of rabbits fed normal chow remained clear, smooth and continuous with no obvious changes (Figure 1D–1F). Hematoxylin and eosin (H&E) was used to stain vascular tissue from sites with obvious plaques in the AS group, and tissues were imaged using 2D ultrasound. Thickened intimae, foam cell deposition, smooth muscle cell proliferation and varying degrees of plaques on rabbit carotid artery intimae were observed (Figure 2A–2C). However, the intimae of the control group were smooth and intact, and no obvious foam cell deposition or smooth muscle cell proliferation was observed (Figure 2D–2F).

### Overview of lncRNA-seq, mRNA-seq and miRNA-seq in the rabbit carotid arteries

Overall, 594,089,506 raw reads were generated, comprising 298,318,754 reads for the carotid atherosclerotic animal models and 295,770,752 reads for the normal controls. From the raw data, reads with adapter, poly-N, and low-quality sequences were removed. In total, we obtained 572,726,694 clean reads in the two different groups, including 288,533,106 reads for the AS samples and 284,193,588 reads for the normal control samples. Furthermore, 33 annotated lncRNAs and 1,622 novel lncRNAs were identified and subsequently analyzed; these lncRNAs included 1,496 lincRNAs (90.4%) and 159 antisense lncRNAs (9.6%) that have not been reported in the past. Sequence length analyses demonstrated that the lncRNA transcripts mostly ranged in length from 200 to 1,000 bp. Furthermore, the length of the ORF in the lncRNA transcripts ranged from 38 to 545, and the number of exons ranged from 2 to 10. Overall, 20,588 protein-coding transcripts were identified. Detected mRNAs were subsequently utilized for further analysis.

As for miRNA-seq in the rabbit carotid arteries, a total of 48,096,306 raw reads were produced and contained 25,287,408 reads from the atherosclerotic rabbit carotid artery samples and 22,808,898 reads from the normal controls. Reads with poly-N sequences, 5’ adapter contamination, poly C, G, T, or A sequences, and low-quality reads were removed as were those without a 3’ adapter or insert tag; finally, 47,233,534 clean reads were obtained from the raw data, including 24,808,717 reads from the AS samples and 22,424,817 reads from the normal control samples. Overall, we found 12,998 known miRNAs and 398 novel miRNAs. These miRNAs were further analyzed.

### DE analysis of AS samples versus normal controls

First, compared with the normal control samples, the AS samples had 73 significantly and DE lncRNA transcripts (q < 0.05, Supplementary Table 1): there were 23 upregulated transcripts and 50 downregulated transcripts in the AS rabbits compared with those in the normal rabbits. Additionally, 50 significantly and DE miRNAs were identified between the two groups (q < 0.05, Supplementary Table 2): 29 DE miRNAs were upregulated, whereas 21 were downregulated. Fragments per kilobase of exon per million fragments mapped (FPKM) were applied to evaluate the mRNA transcript expression level. Finally, we detected 1,099 DE mRNA transcripts (q < 0.05, Supplementary Table 3), including 512 upregulated and 587 downregulated transcripts. The top 10 significantly and DE lncRNAs, miRNAs, and mRNAs based on q-values are summarized in Tables 1–3.

**Table 1:** Top 10 significantly DE lncRNA transcripts between AS and control rabbits.

**transcript_id**	**gene_id**	**gene_location**	**q-value**	**Status**
LNC_001437	XLOC_069865	GL018901:510515-516695	0.00164462	Down
LNC_001325	XLOC_065554	GL018764:170195-171220	0.00164462	Down
LNC_001328	XLOC_065631	GL018765:558169-562504	0.00164462	Up
LNC_000054	XLOC_002863	1:7935208-7970054	0.00164462	Up
LNC_001330	XLOC_065634	GL018765:565577-568665	0.00164462	Up
LNC_000847	XLOC_042092	3:117311930-117314850	0.00164462	Down
LNC_001464	XLOC_071174	GL018980:46374-48378	0.00164462	Up
LNC_001230	XLOC_061964	GL018717:3391415-3396624	0.00164462	Up
LNC_001550	XLOC_073988	GL019367:24423-26582	0.00164462	Up
LNC_001071	XLOC_054118	8:81010850-81196338	0.00164462	Up

**Table 2:** Top 10 significantly DE miRNA transcripts between AS and control rabbits.

**miRNA**	**log2FoldChange**	**q-value**	**status**
ocu-miR-12092-5p	3.5766	1.01E-05	Up
ocu-miR-34a-5p	2.1816	1.01E-05	Up
ocu-miR-411-5p	2.1445	1.01E-05	Up
ocu-miR-204-5p	-2.4656	1.57E-05	Down
ocu-miR-136-3p	1.6557	0.00012739	Up
ocu-miR-424-5p	2.7254	0.00012739	Up
ocu-miR-199a-5p	1.6118	0.00016566	Up
ocu-miR-889-3p	1.9241	0.00019648	Up
ocu-miR-21-5p	1.9544	0.00027826	Up
ocu-miR-450a-5p	2.5477	0.00027826	Up

**Table 3:** Top 10 significantly DE mRNA transcripts between AS and control rabbits.

**transcript_id**	**gene_id**	**gene_name**	**gene_location**	**q-value**	**status**
ENSOCUT00000021509	ENSOCUG00000023515	-	3:38738612-38740667	0.00164462	Down
ENSOCUT00000022366	ENSOCUG00000023019	FOSB	GL019006:121962-124101	0.00164462	Down
ENSOCUT00000010011	ENSOCUG00000010009	EPX	19:30020961-30043919	0.00164462	Down
ENSOCUT00000010716	ENSOCUG00000010718	TIMP1	X:32645153-32703324	0.00164462	Up
ENSOCUT00000004120	ENSOCUG00000004119	GUSB	GL018788:574862-595986	0.00164462	Up
ENSOCUT00000003367	ENSOCUG00000003365	INPP4A	2:93575802-93716698	0.00164462	Down
ENSOCUT00000031476	ENSOCUG00000004314	TRPM1	17:83052800-83126041	0.00164462	Up
ENSOCUT00000027024	ENSOCUG00000023811	-	13:42188650-42189061	0.00164462	Down
ENSOCUT00000008471	ENSOCUG00000008474	CD2	13:47551879-47565052	0.00164462	Down
ENSOCUT00000011353	ENSOCUG00000011355	SCPEP1	19:31071905-31099890	0.00164462	Up

### Construction of the lncRNA-miRNA-mRNA network

RNA transcripts can effectively interact with one another based on the ceRNA hypothesis. RNAs in ceRNA can compete for the same MREs to modulate one another. LncRNAs can absorb miRNAs by binding to miRNAs and subsequently exhibiting a miRNA sponge function. Prediction results from bioinformatic analyses indicated that 746 lncRNAs were targeted by 382 miRNAs; 701 lncRNAs acted as decoys for 369 miRNAs. Based on the lncRNA-miRNA and miRNA-mRNA interaction pairs, a lncRNA-miRNA-mRNA network was constructed. The network consisted of 15,241 lncRNA-miRNA relationship pairs and 42,732 miRNA-mRNA relationship pairs (Supplementary Table 4).

### Functional annotation: GO and KEGG

**Figure 3:**
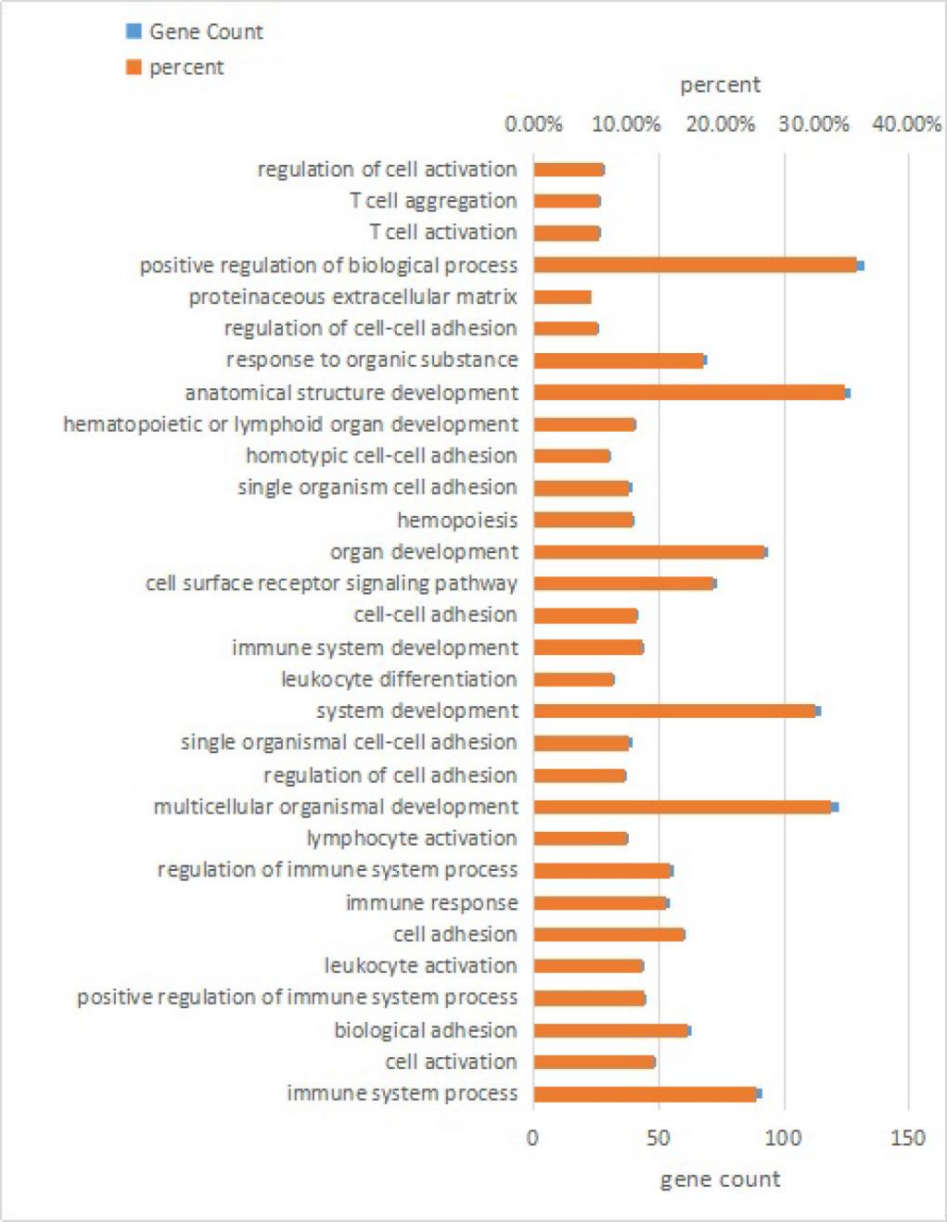
**Top 30 terms of biological process in GO analysis of mRNA genes in the lncRNA-miRNA-mRNA network.**

**Figure 4:**
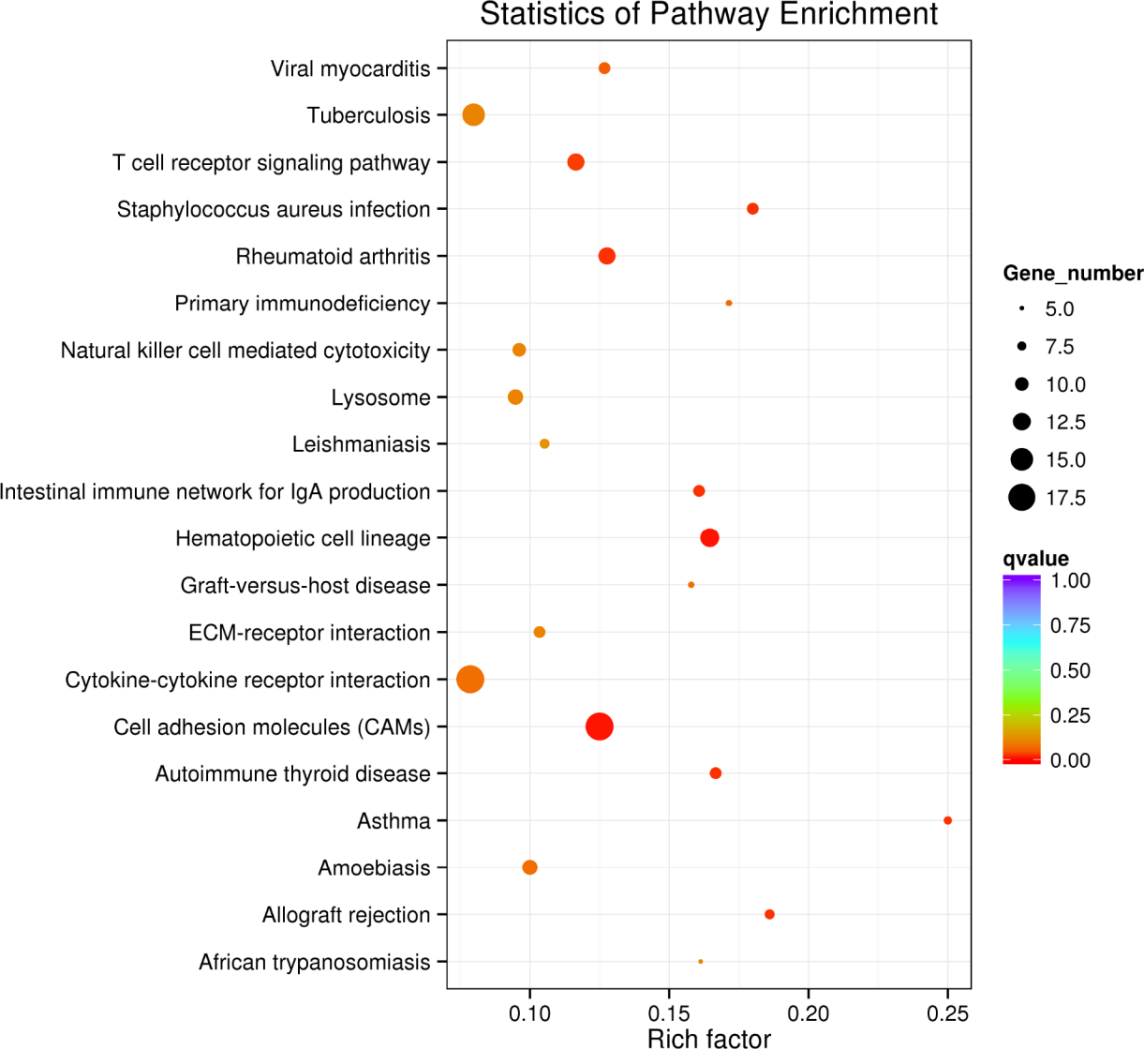
**Top 20 KEGG terms in KEGG analysis of mRNA genes in the lncRNA-miRNA-mRNA network.**

RNA interactions exert various functions expressed in associated mRNA genes. GO and KEGG analyses were conducted on the mRNA genes of the lncRNA-miRNA-mRNA network. Briefly, 424 significant GO-BP terms were observed and enriched (q < 0.05, Supplementary Table 5) in the GO enrichment analysis. The top three terms included immune system process (GO: 0002376), cell activation (GO: 0001775), and biological adhesion (GO: 0022610). Some cognition-related terms were also visualized, such as cell adhesion (GO: 0007155), immune response (GO: 0006955), T cell activation (GO: 0042110), leukocyte activation (GO: 0045321), and cell proliferation (GO: 0008283) (Figure 3). Through the KEGG survey, 33 significant KEGG terms were clustered (p < 0.05, Supplementary Table 6). We observed several cognition-associated terms, including cell adhesion molecules (CAMs) (ocu04514), T cell receptor signaling pathway (ocu04660), cytokine-cytokine receptor interaction (ocu04060), ECM-receptor interaction (ocu04512), and natural killer cell-mediated cytotoxicity (ocu04650) (Figure 4). Overall, the lncRNA-miRNA-mRNA network is involved in the etiopathogenesis of AS from various aspects.

### Analysis of the DE lncRNA-miRNA-mRNA network in AS

**Figure 5:**
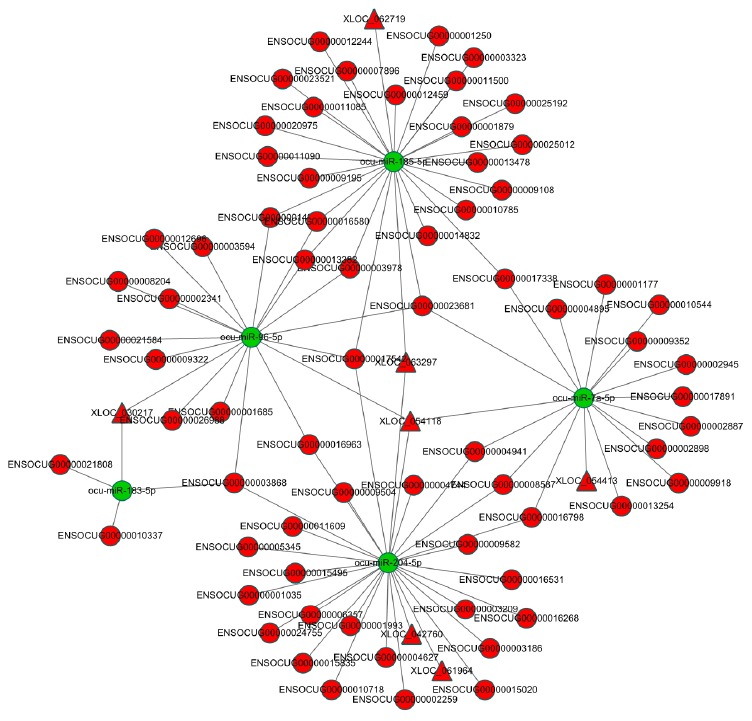
**The dysregulated lncRNA-miRNA-mRNA network in AS rabbits.** The network was based on lncRNA-miRNA and miRNA-mRNA interactions. LncRNA (up in AS rabbits)-miRNA (down in AS rabbits)-mRNA (up in AS rabbits). The red circles represent mRNAs. The red triangles represent lncRNAs. The green circles represent miRNAs.

We further established three restrictions to determine the most likely link between the lncRNA-miRNA-mRNA network and AS. A selective analysis of the lncRNA-mRNA network was conducted, wherein the lncRNAs, miRNAs, and their target genes were significantly dysregulated between the AS rabbits and control group (q < 0.05). Next, the concentrations of the chosen lncRNAs, miRNAs, and their target genes in the carotid arteries of rabbits were determined. The selected dysregulated lncRNA-miRNA-mRNA networks included only one situation (Figure 5): lncRNA (upregulated in AS rabbits)-miRNA (downregulated in AS rabbits)-mRNA (upregulated in AS rabbits) (Supplementary Table 7). These target genes in the selected triple pairs should be related to AS. The filtered lncRNA-miRNA-mRNA network demonstrated high interactions and could be used to explore the biological mechanism of AS. Intriguingly, only four lncRNAs, *XLOC_054118*, *XLOC_030217*, *XLOC_062719* and *XLOC_063297* were identified in our selected data. *CHI3L1*, *SOAT*, *CTSB* and *CAPG* shared the common miRNA *ocu-miR-96-5p* with *XLOC_054118* and *XLOC_030217* as ceRNAs. *CTSB*, *CTSS* and *EDNRA* shared the common miRNA *ocu-miR-185-3p* with *XLOC_062719* and *XLOC_063297* as ceRNAs. The results are listed in Table 4.

**Table 4:** The lncRNA-associated ceRNA network that is most likely involved in AS pathogenesis.

**lncRNA id**	**status**	**q value**	**miRNA id**	**status**	**q value**	**Transcript id**	**mRNA id**	**status**	**gene_name**	**q value**
XLOC_063297	Up	0.001644	ocu-miR- 185-5p	down	0.035479	ENSOCUT00000011082	ENSOCUG00000011085	up	CTSS	0.00164462
						ENSOCUT00000001249	ENSOCUG00000001250	up	EDNRA	0.0367288
XLOC_062719	up	0.023797				ENSOCUT00000016580	ENSOCUG00000016580	up	CTSB	0.00164462
XLOC_054118	Up	0.001644	ocu-miR- 96-5p	down	0.022014	ENSOCUT00000008207	ENSOCUG00000008204	up	SOAT1	0.00164462
						ENSOCUT00000002339	ENSOCUG00000002341	up	CAPG	0.00164462
XLOC_030217	up	0.014355				ENSOCUT00000028603	ENSOCUG00000026988	up	CHI3L1	0.00164462

## DISCUSSION

Atherosclerosis is a chronic inflammatory disorder involving various immune cells at lesion sites and is the main cause of cardiovascular disorders [14]. Efforts have been made to find novel therapeutic targets and biomarkers of AS; however, the focus has been limited to mRNA [15]. Recently, lncRNAs and circRNAs have received attention as novel diagnostic markers for a number of disorders, including carcinomas [16]. In our previous study, we performed a comprehensive analysis of the circRNA expression pattern and circRNA-miRNA-mRNA network in the pathogenesis of AS in rabbits [17]. However, the expression profiles and functions of lncRNAs in AS remain unknown. LncRNAs, miRNAs and mRNAs participate in large-scale ceRNA crosstalk through MREs. This crosstalk has exciting influences for posttranscriptional gene regulation in various physiological and pathophysiological processes.

In this study, we established a rabbit model of the progression and regression of AS through balloon injury followed by an atherogenic diet. Then, we applied a deep RNA-seq analysis to explore the changes in the expression levels of mRNAs, lncRNAs and miRNAs in the progression and regression of AS. First, we detected 73 significantly and DE lncRNA transcripts between the AS samples and the normal control samples with 23 upregulated trasnscripts and 50 downregulated transcripts. Furthermore, 50 significantly DE miRNAs were identified between the two groups: 29 DE miRNAs were upregulated, whereas 21 were downregulated. Finally, we detected 1,099 DE mRNA transcripts, including 512 upregulated transcripts and 587 downregulated transcripts.

Many of these genes are well-known AS-related genes, such as *MARCO*, which is highly expressed in AS patients [18]. Heme oxygenase-1 (HO-1) has been reported to function as an intrinsic protective factor against atherosclerotic lesion formation by inhibiting lipid peroxidation in rabbits [19]. *TLR2*, *4*, and *8* mRNAs are overexpressed in rabbit aortas after a high cholesterol diet, and their expression is correlated with inflammatory and biochemical markers and the further progression of AS [20]. Pryshchep et al. [21] found that *TLR8* expression was very low in the aorta but high in the carotid arteries. Our findings suggested that *TLR2*, *4*, and *8* were also highly expressed in the carotid arteries of atherosclerotic rabbits. VEGFs and other growth factors and cytokines can accelerate neointimal formation and AS by influencing monocyte activation, adhesion, and migration and by enhancing vascular permeability [22]. CXCL8/G31P, an IL-8 analog, can inhibit the formation of atherosclerotic plaques in the coronary artery [23]. Depletion of *miR-34a* facilitated endothelial cell growth and blocked apoptosis in AS by upregulating Bcl-2 [24]. *MiR-497* expression was negatively correlated with apelin protein expression in atherosclerotic lesions [25]. In our experiment, *ocu-miR-34a-5p* and *ocu-miR-497-5p* were also highly expressed in atherosclerotic rabbits.

LncRNAs with sequences similar to those of their target miRNAs serve as ceRNAs to modulate the level of protein-coding genes [10, 11]. According to this ceRNA hypothesis theory, to fully understand the impact of lncRNA-related ceRNA crosstalk on AS, a global miRNA-lncRNA and protein-coding mRNA triple network of AS rabbits and controls was constructed by calculating the Pearson correlation coefficient of miRNA versus lncRNA and that of miRNA versus mRNA. GO and KEGG analyses were performed for the genes in this network and indicated that many enriched terms were associated with AS, including leukocyte activation (GO: 0045321), cell proliferation (GO: 0008283), CAMs (ocu04514) and cytokine-cytokine receptor interaction (ocu04060). The development of atherosclerotic lesions is driven by chronic inflammatory and proliferative processes. Swapnil V. et al. [26] demonstrated that the selective activation of leukocyte GPR120/FFAR4 by n-3 PUFAs, which are atheroprotective in humans, resulted in decreased leukocyte inflammation and AS. The proliferation of vascular smooth muscle cells (VSMCs) in the media layer, stimulated by growth factors from different sources, is an essential step in the formation of plaques [27]. Vascular cell adhesion molecule-1 (VCAM-1), an inflammatory and atherosclerotic marker, is an adhesion molecule abundantly expressed by smooth muscle cells in atherosclerotic lesions and injured arteries. VCAM-1 facilitates monocyte infiltration into atherosclerotic vascular walls and increases VSMC migration, further exacerbating AS [28]. An et al. [29] found that IL-8, a proinflammatory cytokine, interacted with its receptor CXC chemokine receptor 2 on neutrophils leading to the formation of neutrophil extracellular traps to aggravate AS progression in vivo. Thus, we predicted that these lncRNAs may be correlated with AS by regulating gene expression.

**Figure 6:**
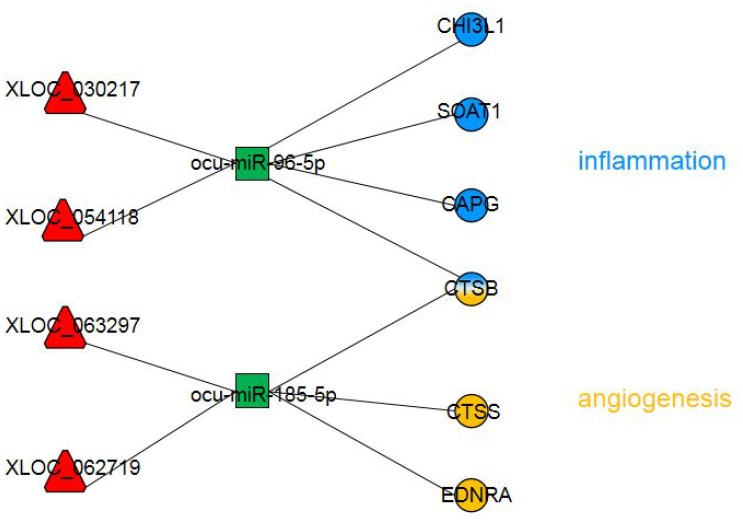
**The dysregulated lncRNA-miRNA-mRNA network most likely involved in AS pathogenesis.** The circles represent mRNAs with blue indicating inflammation and yellow indicating angiogenesis. The red triangles represent lncRNAs. The green squares represent miRNAs.

We applied rigorous restrictions to select the greatest probable lncRNA-related-ceRNA network that participated in the origin, development and changes in AS. First, we selectively analyzed the lncRNA-mRNA network by examining the likely significantly dysregulated RNAs. Next, the concentrations of the selected lncRNAs, miRNAs, and their target genes were determined. Third, the target genes in the chosen triple pairs should be related to AS. Finally, six qualified triple pairs were selected (Figure 6). We discovered that *XLOC_054118* and *XLOC_030217* were ceRNAs of *ocu-miR-96-5p* targeting *CHI3L1*, *SOAT1*, *CAPG* and *CTSB*. Macrophages, which are the primary cells of innate immunity, have vital effects in each stage of AS [30]. CHI3L1 has been detected to to be secreted from differentiated macrophages in early-stage AS lesions [31]. *SOAT1-/-* regulated the suppression of inflammatory molecules, including TNF-α, IL-6 and IL-1b, and alleviated the development of atherosclerotic lesions, exhibiting a positive effect [32]. Association analysis revealed that the rs6886 polymorphism in *CAPG* was linked with carotid intima-media thickness, which is a validated marker of AS; this mechanism could be associated with differential macrophage migration ability and the inflammation process [33]. Levels of *CTSB* mRNA and protein in atherosclerotic lesions of apoE-deficient mice have been observed to increase; *CTSB* immunoreactivity levels were highest in areas next to the lumen and in macrophages [34]. Interestingly, we found that the above four genes are associated with macrophages and involved in the inflammatory response, which further affects AS. Pordzik J [35] reported that as a platelet miRNA, *miR-96* could be exploited as a biomarker in inflammatory disorders, such as cardiovascular disorders. Therefore, to the best of our knowledge, this is the first report that implies the lncRNAs *XLOC_054118* and *XLOC_030217* function as molecular sponges of the miRNA *ocu-miR-96-5p* to upregulate macrophages and inflammation-associated gene expression consequently participating in AS. Further experiments should be conducted to acquire detailed information on the pathway of genes and function of this network.

We also found that *XLOC_062719* and *XLOC_063297* were ceRNAs of *ocu-miR-185-3p* targeting *CTSB*, *CTSS* and *EDNRA*. Inflammation and angiogenesis broadly interact by sharing several cellular and molecular mediators. A key molecule in both processes is *CTSB* [36]. In vivo, *CTSB* inhibition retards tumorigenesis and vascular growth [37]. In contrast, Im et al. [37] found *CTSB* to be antiangiogenic in vitro, limiting endothelial tube formation through the degradation of endogenous *VEGF-A*. *EDNRA* mediates vasoconstriction and cell proliferation [38]. Research on *EDNRA* gene polymorphism showed that rs5333 was significantly associated with mean carotid intima-media thickness in men [39]. Microvessels may increase atherosclerotic plaque growth and lesion instability [40]. Recent studies have suggested that *CTSS* may contribute to the degradation of arterial ECM, which provides a path for neovessel growth [41]. *CTSS* deficiency reduces microtubule formation in vitro and microvessel growth in vivo. Interestingly, we found that these three genes are associated with angiogenesis and proliferation, which further affects AS. K Shan et al. showed that *miR-185-5p* is a posttranscriptional regulator of inflammation. *RNCR3* is significantly upregulated, which alleviates *miR-185-5p* repression, thereby upregulating the level of the *miR-185-5p* target gene, *KLF2*. This regulatory loop maintains an associated balance in endothelial function to resist proatherogenic stress [42]. Therefore, we hypothesized that *XLOC_062719* and *XLOC_063297* functioned as molecular sponges of the miRNA *ocu-miR-185-5p* to upregulate angiogenesis and proliferation-associated gene expression, consequently participating in AS. Additional research is required to fully reveal the pathway of targeted genes and the involvement of these three lncRNAs in regulating angiogenesis in AS.

### Limitations

The results of the present study have not been experimentally validated and require confirmation. Additionally, the sample sizes were also small (three control samples and three AS samples).

### Future directions

As more functional lncRNAs are identified in AS, lncRNAs will be increasingly and progressively used as diagnostic and therapeutic markers. An RNAi therapeutic strategy for AS could be to interfere with the lncRNA expression program that underlies AS [15]. Its potential for treating AS is supported by the discovery of lncRNAs and their effect on gene expression profiles. The use of lncRNA strategies could open novel therapeutic avenues for AS. However, large groups of essential, important lncRNAs remain unreported due to technical or cognitive limitations. More research is required to identify the novel mechanisms and functions of lncRNAs.

## CONCLUSIONS

This is the first study to comprehensively analyze dysregulated lncRNA-miRNA-mRNA networks in AS. We showed that functional lncRNAs are involved in regulating carotid atherosclerotic rabbit models. In the network, *XLOC_054118* and *XLOC_030217* upregulate the *CHI3L1*, *SOAT*, *CTSB* and *CAPG* genes by competitively binding to the miRNA *ocu-miR-96-5p*. Furthermore, *XLOC_062719* and *XLOC_063297* upregulate the *CTSS*, *CTSB* and *EDNRA* genes by competitively binding to the miRNA *ocu-miR-185-5p*. These four lncRNAs could act as prospective clinical markers associated with the development of AS. However, additional work is needed to uncover the underlying mechanisms of lncRNAs in AS.

## MATERIALS AND METHODS

### Animals

Six New Zealand white adult male rabbits (2.5-3.5 kg) were purchased from the Model Animal Center of the Second Affiliated Hospital of Harbin Medical University. Fan et al. reported the feasibility and validity of rabbit models to study human AS [43]. All animals received humane care in compliance with the Guidelines for the Care and Use of Laboratory Animals. All procedures were conducted on the basis of the guidelines established by the National Institutes of Health. Our study protocol was approved by the Medical Ethics Committee on Animal Research of the 2^nd^ Affiliated Hospital of Harbin Medical University (Ethics No. KY2016-090).

### Carotid atherosclerotic animal models

In order to induce carotid atherosclerosis, three rabbits were fed an atherogenic diet including 10% lard (Shandong Shiyuantianjiaji Factory), 3% yolk powder (Shandong Shiyuantianjiaji Factory), and 1% cholesterol (Shanghai Lanji Technology) combined with basal feed as the AS group. After one week, the three rabbits were intramuscularly anesthetized with ketamine (35 mg/kg), xylazine (5 mg/kg) and acepromazine (0.75 mg/kg). Following the methods of a previous study [44], the right common carotid arteries (CCA) were injured with a 2F Fogarty balloon catheter (Boston Scientific, Temecula, California), which was gently advanced into the CCA through the external carotid artery. The balloon was gradually inflated at 2 atm and retracted. This procedure was repeated three times in each rabbit, and anesthesia was maintained by isoflurane inhalation. Then, the balloon catheter was removed, the incision was closed with a suture, and the rabbits were allowed to recover while on the atherogenic diet. The carotid intimae of these rabbits were monitored weekly by two-dimensional ultrasound until significant plaques appeared at the 12^th^ week. Three rabbits were sacrificed by air embolism. From the right carotid artery with the most obvious plaque (based on ultrasonic detection), the carotid atherosclerotic plaque and intima were excised and immediately preserved in liquid nitrogen. Three age-matched rabbits were fed a basal diet served as the control group, and the same tissue samples of the carotid artery as in the AS group were excised at the 12^th^ week. The obtained arteries were numbered, marked and fixed with a 4% paraformaldehyde fixative and embedded in paraffin for subsequent H&E staining. Continuous cross-sections with a thickness of 3 μm were stained with H&E and observed by light microscopy (Olympus, BX41, Tokyo, Japan).

### RNA extraction and library preparation for sequencing

NEBNext® Ultra™ Directional RNA Library Prep Kit for Illumina® (NEB, Ipswich, MA, USA) was used to prepare lncRNA and mRNA libraries following the manufacturer’s recommendations. Paired-end reads of 150 bp were generated with an Illumina HiSeq X platform. After quality control, paired-end clean reads were mapped to the reference genome (OryCun2.0) by TopHat v2.0.9. Cufflinks (http://cufflinks.cbcb.umd.edu/) was used to assemble and annotate the transcripts. According to the annotation of the genome sequence (OryCun2.0), known lncRNAs and mRNAs were identified. We used the remaining transcripts to screen for putative lncRNAs according to the following criteria: (1) exon number ≥ 2; (2) sequencing coverage ≥ 3; (3) length ≥ 200 bp; and (4) identification in more than one sample. The transcripts meeting the above criteria were further filtered by removing known non-lncRNA transcripts. Next, CPC (0.9-r2) [45] and Pfam-scan (v1.3) [46] were applied to evaluate the transcripts that passed the filters for coding potential. Only transcripts without coding potential were classified as novel lncRNAs.

NEBNext® Ultra™ Directional RNA Library Prep Kit for Illumina® (NEB, Ipswich, MA, USA) was used to prepare small RNA library preparation following the manufacturer’s recommendations. Single-end reads of 50 bp were generated with an Illumina HiSeq 2500 platform. After quality control, the clean reads were aligned to a reference sequence (OryCun2.0) using Bowtie [47]. Mapped reads were used to identify known miRNAs applying miRBase 20.0 [48]. MiREvo [49] and miRDeep2 [50] were utilized to predict novel miRNAs by investigating characteristic hairpin structures, minimum free energy and Dicer cleavage sites.

### Identification and clustering analyses of DE lncRNAs, miRNAs and mRNAs

We used the DE Seq R package (1.8.3) to identify DE lncRNAs, microRNAs and mRNAs (Benjamini & Hochber method corrected p-value <0.05) for AS rabbits and controls. We applied the R package “pheatmap” to analyze the expression of lncRNAs, microRNAs and mRNAs with unsupervised hierarchical clustering. The expression of each RNA type was normalized for unsupervised hierarchical clustering. Expressions of lncRNA and mRNA were normalized using the following formula: FPKm =lg_10_ FPKM + 1. Expression of miRNA was normalized with the following formula: TPm =lg_10_ TPM + 1 (TPM, transcripts per kilobase million). Next, the degree of similarity between the expression profiles of samples was measured using the Euclidean distance (using the R package, “complete”).

### Prediction of lncRNA and miRNA target genes

Correlating expression levels between lncRNAs and mRNAs were used to assess potential trans roles of lncRNAs (acting on non-neighboring genes). Based on the expression correlation coefficient (Pearson correlation absolute value greater 0.8), we examined the trans role of lncRNAs in coding genes. To predict miRNA targets, we searched for targets in the 3′ UTR of gene models. For genes lacking a predicted 3′ UTR, regions 1,000 bp downstream of the stop codon were included. Miranda was conducted to perform the prediction (free energy <10 kcal/mol and score >140) [51].

### GO and KEGG enrichment analyses

We utilized the GO database to analyze the lncRNA-miRNA-targeted genes. GO analysis was conducted with the GO-seq R package. Briefly, 424 GO-BP terms were significantly enriched (q < 0.05). As a database resource, KEGG can comprehend high-level utility functions of biological systems. KOBAS software was used to check the statistical enrichment of lncRNA target or dysregulated genes in the KEGG pathways. Thirty-three KEGG terms were significantly enriched (q < 0.05).

### Construction of the lncRNA-miRNA-mRNA network

LncRNAs predicted to function as miRNA targets or decoys by Fan's methods were selected [52] to construct the lncRNA-miRNA-mRNA network. Next, we calculated the Pearson correlation coefficient value between a microRNA and its target mRNA and subsequently selected strongly correlated miRNA-mRNA pairs (with values greater than 0.8 or less than -0.8) to define the miRNA-mRNA relationships. To construct the lncRNA-miRNA-mRNA network, each DE RNA hub must be in either a miRNA-mRNA pair or a lncRNA-miRNA pair. The hubs in the lncRNA-miRNA-mRNA network consisted of microRNAs, lncRNAs acting as microRNA decoys, lncRNAs acting as microRNA targets, and mRNAs acting as microRNA targets. We used Cytoscape (version 3.4.0) [53] to visualize this network.

## Supplementary Material

Supplementary Tables

Supplementary Table 1

Supplementary Table 2

Supplementary Table 3

Supplementary Table 4

Supplementary Table 5

Supplementary Table 6

Supplementary Table 7
