# Supplementary material for: Functional lncRNA-miRNA-mRNA networks in rabbit carotid atherosclerosis
Source: Aging (Albany NY). 2020 Feb 11;12(3):2798–813. doi: 10.18632/aging.102778 (PMC7041763; doi:10.18632/aging.102778)
Supplement: Supplementary Tables [file aging-12-102778-s005..pdf]

SUPPLEMENTARY TABLES

Please browse Full Text version to see the data of Supplementary Tables 1–7

Supplementary Table 1. Significantly DE lncRNA transcripts between AS and control rabbits.

Supplementary Table 2. Significantly DE miRNA transcripts between AS and control rabbits.

Supplementary Table 3. Significantly DE mRNA transcripts between AS and control rabbits.

Supplementary Table 4. LncRNA-miRNA and miRNA-mRNA interaction pairs.

Supplementary Table 5. GO enrichment analysis of the lncRNA-miRNA-mRNA-targeted genes.

Supplementary Table 6. KEGG enrichment analysis of the lncRNA-miRNA-mRNA-targeted genes.

Supplementary Table 7. The lncRNA-miRNA-mRNA network. LncRNA (up in AS rabbits)-miRNA (down in AS rabbits)-mRNA (up in AS rabbits).
